# Acute Illnesses and Injuries Related to Total Release Foggers — 10 States, 2007–2015

**DOI:** 10.15585/mmwr.mm6704a4

**Published:** 2018-02-02

**Authors:** Ruiling Liu, Walter A. Alarcon, Geoffrey M. Calvert, Kathleen G. Aubin, John Beckman, Karen R. Cummings, Lucia S. Graham, Sheila A. Higgins, Prakash Mulay, Ketki Patel, Joanne B. Prado, Abby Schwartz, Derry Stover, Justin Waltz

**Affiliations:** ^1^National Institute for Occupational Safety and Health, CDC; ^2^Louisiana Department of Health; ^3^California Department of Public Health; ^4^Public Health Institute, Oakland, California; ^5^New York State Department of Health; ^6^California Department of Pesticide Registration; ^7^North Carolina Division of Public Health; ^8^Florida Department of Health; ^9^Texas Department of State Health Services; ^10^Washington State Department of Health; ^11^Michigan Department of Health and Human Services; ^12^Nebraska Department of Health and Human Services; ^13^Oregon Health Authority.

Total release foggers (TRFs) (also known as “bug bombs”) are pesticide products often used indoors to kill insects. After an earlier report found that TRFs pose a risk for acute illness (*1*), the Environmental Protection Agency required improved labels on TRFs manufactured after September 2012 (*2*). To examine the early impact of relabeling, the magnitude and characteristics of acute TRF-related illness were evaluated for the period 2007–2015. A total of 3,222 TRF-related illnesses were identified in 10 participating states, based on three data sources: Sentinel Event Notification System for Occupational Risk–Pesticides (SENSOR) programs, the California Department of Pesticide Regulation (CDPR) program, and poison control centers (PCCs) in Florida, Texas, and Washington. No statistically significant decline in the overall TRF-illness incidence rate was found. Failure to vacate treated premises during application was the most commonly reported cause of exposure. To reduce TRF-related illness, integrated pest management strategies (*3*) need to be adopted, as well as better communication about the hazards and proper uses of TRFs. Redesigning TRFs to prevent sudden, unexpected activation might also be useful.

Acute TRF-related illnesses were identified from the SENSOR programs in 10 participating states (2007–2015),* CDPR^†^ (2007–2014), and PCCs in Florida, Texas, and Washington (2007–2015). Complete PCC data were unavailable from the other seven states with SENSOR programs. Cases meeting all of the following criteria were included: exposure to TRFs with known active ingredients, at least two signs or symptoms related to or possibly related to TRF exposure, and no involvement of suicide or intentional harm to others. A total of 3,222 unique cases were identified.^§^ Cases were categorized as definite, probable, or possible based on case-level evidence.^¶^ The magnitude, trends, and characteristics of acute TRF-related illnesses were assessed. Incidence rates were calculated using U.S. Census standard population estimates as denominators (*4*). Poisson regression analyses were conducted to estimate incidence rate ratios (IRR) during 2013 (the first full year after label improvement when many TRF products on store shelves likely still had the old labels) and 2014–2015 (the period after label improvement when most TRF products likely had new labels) compared with 2007–2012 (the period before label improvements) for all cases and by reported causes of exposure, controlling for state to adjust for discordance in missing data across states. Stepwise logistic regression analysis was conducted to explore reported causes associated with more severe illness** (high or moderate versus low severity), adjusting for age, sex, and preexisting health conditions.

Overall, 3,573 cases were identified, including 1,843 from the SENSOR and CDPR programs and 1,730 from PCCs in Florida, Texas, and Washington (Table 1); 351 cases were identified from both the SENSOR programs and PCCs in Florida, Texas, or Washington, resulting in a total of 3,222 unique cases. Among cases from the SENSOR and CDPR programs, 87% were reported to the programs by PCCs; 6% were classified as definite, 20% as probable, and 74% as possible. After combining unique cases from the three data sources, the overall incidence rates in the 10 states during 2007–2012, 2013, and 2014–2015 were 27.0, 26.3, and 29.5 per 10 million population, respectively. The adjusted incidence rate did not change in 2013 or 2014–2015, compared with 2007–2012 (Table 2).

**TABLE 1:** Selected characteristics for acute illnesses and injuries related to total release foggers (TRFs) reported to the Sentinel Event Notification System for Occupational Risk (SENSOR)–Pesticides program, the California Department of Pesticide Registration (CDPR), and poison control centers (PCCs) — 10 states, 2007–2015

Characteristic	SENSOR and CDPR (n = 1,843)		PCCs (n = 1,730)		Total* (N = 3,222)	
	No.	(%)	No.	(%)	No.	(%)
**Reporting state (yrs data available)**						
Texas (2007–2015)	38	(2.1)	912	(52.7)	915	(28.4)
Florida (2007–2015)	301	(16.3)	582	(33.6)	658	(20.4)
North Carolina (2007–2015)	467	(25.3)	—	—	467	(14.5)
Michigan (2007–2015)	255	(13.8)	—	—	255	(7.9)
Washington (2007–2015)	107	(5.8)	236	(13.6)	252	(7.8)
California (2007–2014)^†^	234	(12.7)	—	—	234	(7.3)
Louisiana (2007–2015)	198	(10.7)	—	—	198	(6.2)
New York (2007–2014)	166	(9.0)	—	—	166	(5.2)
Oregon (2007–2013)	55	(3.0)	—	—	55	(1.7)
Nebraska (2007–2014)	22	(1.2)	—	—	22	(0.7)
**Year**						
2007	155	(8.4)	159	(9.2)	248	(7.7)
2008	229	(12.4)	161	(9.3)	350	(10.9)
2009	273	(14.8)	195	(11.3)	407	(12.6)
2010	231	(12.5)	236	(13.6)	402	(12.5)
2011	227	(12.3)	179	(10.4)	348	(10.8)
2012	247	(13.4)	223	(12.9)	453	(14.1)
2013	183	(9.9)	202	(11.7)	372	(11.6)
2014	163	(8.8)	169	(9.8)	325	(10.1)
2015	135	(7.3)	206	(11.9)	317	(9.8)
**Case status**						
Definite	105	(5.7)	—	—	105	(3.3)
Probable	366	(19.9)	—	—	366	(11.4)
Possible	1,372	(74.4)	—	—	1,372	(42.6)
Not evaluated	—	—	1,730	(100.0)	1,379	(42.8)
**Age group (yrs)**						
0–5	95	(5.2)	93	(5.4)	173	(5.4)
6–12	71	(3.9)	84	(4.9)	141	(4.4)
13–17	58	(3.2)	42	(2.4)	90	(2.8)
18–59	1,292	(70.1)	1,100	(63.6)	2,131	(66.1)
≥60	253	(13.7)	245	(14.2)	456	(14.2)
Unknown adult (≥20)	—	—	144	(8.3)	144	(4.5)
Unknown	74	(4.0)	22	(1.3)	87	(2.7)
**Sex**						
Female	1,017	(55.2)	1,007	(58.2)	1,818	(56.4)
Male	789	(42.8)	713	(41.2)	1,362	(42.3)
Unknown	37	(2.0)	10	(0.6)	42	(1.3)
**Location of exposure**						
Private residence	1,570	(85.2)	1,641	(94.9)	2,954	(91.7)
Nonmanufacturing commercial site	58	(3.1)	54	(3.1)	99	(3.0)
Other^§^	88	(4.8)	31	(1.8)	106	(3.3)
Unknown	127	(6.9)	4	(0.2)	63	(2.0)
**Work-related exposure**						
Yes	162	(8.8)	52	(3.0)	176	(5.5)
No	1,506	(81.7)	1,674	(96.8)	2,946	(91.4)
Unknown	175	(9.5)	4	(0.2)	100	(3.1)
**Sites of signs and symptoms** ^¶^						
Respiratory	1,423	(77.2)	1,021	(59.0)	2,182	(67.7)
Gastrointestinal	755	(41.0)	997	(57.6)	1,584	(49.2)
Neurologic	652	(35.4)	421	(24.3)	945	(29.3)
Cardiovascular	289	(15.7)	210	(12.1)	460	(14.3)
Ocular	272	(14.8)	229	(13.2)	439	(13.6)
Dermatologic	237	(12.9)	215	(12.4)	406	(12.6)
**Severity**						
Fatal	2	(0.1)	2	(0.1)	4	(0.1)
High	17	(0.9)	8	(0.5)	21	(0.7)
Moderate	352	(19.1)	385	(22.3)	669	(20.7)
Low	1,472	(79.9)	1,335	(77.2)	2,528	(78.5)
**Active ingredients involved**						
Pyrethroid	1,493	(81.0)	1,298	(75.0)	2,510	(77.9)
Pyrethrin	604	(32.8)	299	(17.3)	773	(24.0)
Organophosphate	120	(6.5)	80	(4.6)	162	(5.0)
Other**	82	(4.5)	65	(3.8)	140	(4.4)
**Reported causes of exposure** ^††^						
Failure to vacate premises during application	300	(16.3)	201	(17.5)	475	(16.6)
Early reentry	282	(15.3)	150	(13.1)	423	(14.8)
Inability to vacate before TRF discharge	187	(10.2)	128	(11.1)	307	(10.7)
Inadequate ventilation	192	(10.4)	86	(7.5)	263	(9.2)
Sprayed in face or at close range	149	(8.1)	115	(10.0)	258	(9.0)
Excessive fogger use^§§^	154	(8.4)	22	(1.9)	159	(5.5)
Failure to notify others	101	(5.5)	63	(5.5)	146	(5.1)
Discharge by child aged <13 years	70	(3.8)	61	(5.3)	125	(4.4)
Using TRF as spot spray	58	(3.2)	39	(3.4)	91	(3.2)
Unintentional discharge	24	(1.3)	22	(1.9)	45	(1.6)
Other	163	(8.8)	76	(6.6)	225	(7.9)
Unknown	287	(15.6)	210	(18.3)	485	(16.9)
Not evaluated	—	—	582	(33.6)	357	(11.1)

* SENSOR programs in Florida, Texas, and Washington identified 351 cases that were also reported to PCCs. These cases were counted only once in the total; as such, the case numbers under total might be not equal to the sum of the case numbers under SENSOR and CDPR and PCC.

^†^ Among the 234 cases reported by California, 15 were by CDPH via the SENSOR program, 232 by CDPR, and 13 by both.

^§^ The most common other locations were vehicles (21), manufacturing facilities (20), and residential institutions (14).

^¶^ A patient could have signs or symptoms involving multiple sites.

** Other active ingredients were those that did not involve pyrethroids, pyrethrins, or organophosphates. The two most common other active ingredients were N-methyl carbamates (62) and chlorinated hydrocarbons (28). A person could be exposed to a TRF product with multiple active ingredients, thus the sum of cases by active ingredient types exceeds the total number of cases. Among the 3,222 cases, 358 were exposed to more than one of the four categories of active ingredients, and fewer than 5% were exposed to both TRF and non-TRF pesticide products.

^††^ Exposure narratives were not available for cases provided by Florida PCCs; as such, it was not possible to identify causes of the 357 cases reported to Florida PCCs but not to the SENSOR program. The denominators were the total number of cases with reported causes of exposure, except for the category “not evaluated,” for which the denominator was the number of all cases. In addition, a case could have had more than one reported cause of exposure, thus the sum of the rows exceeds the total. The three most commonly reported causes of exposure under the “other” category were contaminated food, drink, utensils, or residue on furniture or surfaces (64); drift (usually from a neighboring apartment unit) (53); and equipment failure (34).

^§§^ Case narratives indicated more foggers were used than necessary. The label specifies that “one 6-oz can treats up to 5,000 ft^3^ of unobstructed space (25 ft x 25 ft x 8 ft ceiling),” and the label cautions, “Do not use more than one fogger per room.”

**TABLE 2:** Incidence of acute total release fogger (TRF)–related illnesses, by reported causes of exposure — 10 states,* 2007–2012, 2013, and 2014–2015

Reported causes of exposure^†^	2007–2012 (before label improvement)		2013 (first year after label improvement)				2014–2015 (after full implementation of label improvement)			
	No. of cases	Observed rate^§^	No. of cases	Observed rate^§^	Adjusted IRR (95% CI)^¶^	p*-*value	No. of cases	Observed rate^§^	Adjusted IRR (95% CI)^¶^	p-value
**Total**	**2,208**	**27.0**	**372**	**26.3**	**0.97 (0.84–1.12)**	**0.704**	**642**	**29.5**	**0.91 (0.81–1.02)**	**0.111**
Failure to vacate premises during application	263	4.0	57	4.9	1.20 (0.91–1.59)	0.200	123	7.0	1.39 (1.12–1.71)	0.002
Early reentry	262	4.0	47	3.9	0.98 (0.66–1.45)	0.915	75	4.3	0.89 (0.64–1.23)	0.473
Inability to vacate before TRF discharge	188	3.3	36	3.0	0.98 (0.74–1.30)	0.872	52	3.2	0.86 (0.67–1.10)	0.229
Inadequate ventilation	153	2.6	23	2.1	0.82 (0.44–1.55)	0.549	71	4.5	1.36 (0.89–2.07)	0.155
Sprayed on face or at close range	151	2.6	28	2.3	0.91 (0.57–1.45)	0.685	41	2.6	0.92 (0.62–1.38)	0.700
Excessive fogger use	121	2.2	19	1.8	0.98 (0.65–1.48)	0.934	12	0.9	0.43 (0.26–0.71)	0.001
Failure to notify others	93	1.8	17	1.6	1.05 (0.75–1.48)	0.762	27	1.7	0.77 (0.57–1.03)	0.074
Discharge by child aged <13 years	76	1.8	12	1.2	0.71 (0.39–1.30)	0.269	26	2.3	0.95 (0.62–1.45)	0.797
Use of TRF as spot spray	58	1.2	11	1.4	0.90 (0.50–1.64)	0.735	14	1.1	0.87 (0.51–1.50)	0.614
Unintentional discharge	24	1.0	8	0.9	1.02 (0.62–1.66)	0.950	10	0.9	1.03 (0.67–1.57)	0.906

**Abbreviations:** CI = confidence interval; IRR = incidence rate ratio; TRF = total release fogger.

* Acute TRF-related illnesses were identified during 2007–2015 from the Sentinel Event Notification System for Occupational Risk (SENSOR)–Pesticides programs in 10 participating states (California, Florida, Louisiana, Michigan, Nebraska, New York, North Carolina, Oregon, Texas, and Washington) and from the California Department of Pesticide Regulation (CDPR) program and poison control centers (PCCs) in Florida, Texas, and Washington.

^†^ Total includes all 3,222 reported cases of acute TRF-related illness. However, for specific reported causes of exposure, Florida cases were excluded because case narratives were not available for any of the 357 Florida PCC cases that were not reported to the SENSOR program. In addition, although the Florida SENSOR program has case narratives available, a trend analysis using Florida data was unreliable because of a sharp drop in reported cases beginning in 2012 that was related to resource limitations. This does not affect the trend analysis for the total because the overall trend includes all Florida PCC cases, and there is no evidence of concerns that would affect reporting to the PCCs (75% [225 of 301] of Florida SENSOR cases that were ascertained by the PCCs and then reported to SENSOR).

^§^ Per 10 million population, based on U.S. Census standard population estimates. https://www2.census.gov/programs-surveys/popest/tables.

^¶^ IRR and 95% confidence intervals were estimated by Poisson regression analysis, controlling for state to adjust for discordance in missing data among states and correcting for overdispersion (greater variability than expected based on Poison distribution). Incidence rate during 2007–2012 was the denominator. For each reported cause, a separate Poisson regression analysis was conducted.

Five percent of cases occurred in children aged 0–5 years and 14% in adults aged ≥60 years (Table 1); the median age was 40 years. Approximately 56% occurred in females; 92% of exposures happened in private residences, and 91% were not work-related. Respiratory signs and symptoms (cough, upper respiratory pain or irritation, and dyspnea) and gastrointestinal signs and symptoms (vomiting, nausea, and abdominal pain or cramping) were the most commonly reported. Severity was classified as low, moderate, and high for 78%, 21%, and 0.7% of the illnesses, respectively. Four (0.1%) cases were fatal. Approximately 93% of cases involved exposure to the TRF active ingredients pyrethroid (78%) or pyrethrin (24%). The most commonly reported causes of exposure were failure to vacate treated premises during application, early reentry into treated premises, inability to vacate treated premises before TRF discharge, and inadequate ventilation of treated premises; approximately 4% of cases were caused by TRF discharge by children aged <13 years (Table 1). Incidence rates associated with failure to vacate premises during application increased during 2014–2015 compared with 2007–2012 (adjusted IRR = 1.39, p = 0.002), whereas rates related to excessive fogger use (i.e., using more foggers than necessary) decreased (adjusted IRR = 0.43, p = 0.001) (Table 2). Moderate or high severity illness were more common among males, persons aged >60 years, those with preexisting asthma, and those who failed to vacate premises during application, or who were exposed to excessive TRFs (Table 3).

**TABLE 3:** Characteristics related to high or moderate severity of total release fogger–related illnesses reported to the Sentinel Event Notification System for Occupational Risk (SENSOR)–Pesticides program and the California Department of Pesticide Registration — 10 states, 2007–2015

Characteristic	No. of cases	Odds ratio (95% CI)*	p-value
**Age group (yrs)**			
0–5	95	0.48 (0.24–0.95)	0.034
6–12	71	0.54 (0.26–1.11)	0.092
13–17	58	0.65 (0.31–1.37)	0.260
18–59	1,292	Referent	—
≥60	251	1.70 (1.25–2.32)	0.001
Unknown	74	0.32 (0.13–0.82)	0.017
**Sex**			
Female	1,015	0.75 (0.59–0.96)	0.020
Male	789	Referent	—
Unknown	37	1.41 (0.51–3.88)	0.51
**Preexisting asthma**			
Yes	139	2.50 (1.71–3.65)	<0.001
No/Unknown	1,702	Referent	—
**Failure to vacate premises during application**			
Yes	300	1.57 (1.17–2.11)	0.003
No/Unknown	1,541	Referent	—
**Excessive fogger use**			
Yes	152	1.54 (1.04–2.27)	0.031
No/unknown	1,689	Referent	—
**Early reentry**			
Yes	282	0.58 (0.39–0.84)	0.005
No/Unknown	1,559	Referent	—

**Abbreviation:** CI = confidence interval.

* Odds ratios were estimated using step-wise logistic regression analysis: entry p-value = 0.10 and stay p-value = 0.15. The outcome of interest was high or moderate severity illness compared with low severity illness, and independent variables included age group, sex, three preexisting conditions (pregnancy, preexisting asthma, and history of allergies), and the top 10 reported causes of exposure (failure to vacate premises during application, early reentry, unable to vacate before total release fogger [TRF] discharge, inadequate ventilation, sprayed on face or at close range, excessive TRF use, failure to notify others, within reach of child, using TRF as spot spray, and unintentional discharge); only variables selected for the final regression model are presented in the table. Data from poison control centers in Florida, Texas, and Washington were not included because they did not provide detailed information for this analysis.

## Discussion

A previous study identified 466 acute TRF-related illnesses in eight states during 2001–2006 (*1*) for a crude average annual incidence rate of seven cases per 10 million population. This study identified 3,222 cases in 10 states during 2007–2015, with an average annual incidence rate of 27 per 10 million population. This increase likely resulted from including all PCC cases from Florida, Texas, and Washington and conducting a more comprehensive search for TRF-related cases in the SENSOR database. The increase might also partly result from increased TRF use and improved case ascertainment in recent years.

The Environmental Protection Agency required registrants of all TRFs manufactured after September 2012 to adopt improved labels that use pictures to illustrate some instructions and precautions and emphasize actions such as vacating treated premises for at least 2 hours, ventilating treated areas before reentry for an additional 2 hours or until no odor is detected, and not using more foggers than necessary. However, exposure narratives from case reports suggested that many users did not follow or read label instructions. Although many users left the treated area or room, they did not leave the treated premises as specified by the label. Early reentry usually involved entering treated premises shortly after application, often to turn off smoke alarms or retrieve pets or forgotten items. Some users were exposed when they entered premises to initiate ventilation. TRF labels do not provide guidance on how to minimize exposure when initiating ventilation. Some users ventilated treated premises for the recommended length of time or longer, but still became ill, suggesting that ventilation might be inadequate or the recommended period might be insufficient to fully eliminate TRF residuals before occupancy. Some were sprayed in the face or at close range because of nozzle malfunction or inappropriate TRF activation (e.g., pointing the nozzle in the wrong direction), suggesting a need for better nozzle designs and a label picture showing how to appropriately set off a TRF.

The reason that the overall illness incidence rate did not decline during 2014–2015 is unknown. Some TRFs used during 2014–2015 might have had old labels, or more time might be needed for the protective effects of the revised labels to be realized. Many users might not have read or followed label instructions. However, incidence rates associated with excessive fogger use did decline, suggesting that simplified label statements and pictures addressing this risk factor might have been effective.

Early reentry likely led to brief exposure to TRF and more commonly caused low severity illnesses, whereas failure to vacate treated premises or excessive fogger use likely resulted in longer or higher concentration exposures and more commonly caused moderate or high severity illnesses. Preexisting asthma was associated with moderate or high severity illnesses, indicating that a warning message for persons with asthma might be necessary on the labels. Although a previous Environmental Protection Agency assessment reported no association between pyrethrin or pyrethroid exposure and asthma (*5*), a recent study found that among persons with acute pesticide-related illness, those with pyrethrin or pyrethroid exposures were significantly more likely to have asthmatic symptoms than were those with other pesticide exposures (*6*).

The findings in this report are subject to at least five limitations. First, because reporting to the surveillance systems and PCCs is passive, and because many persons with low severity cases do not seek medical care, acute TRF-related cases were likely underreported. Second, some cases might be false positives because many of the reported symptoms are not specific to TRF exposure and might have been caused by unrelated factors or conditions. Third, because the number of TRF users or another proxy for TRF users were not available, the overall population in the 10 states was used as the denominator to estimate incidence rates. Trends in incidence rates might be different if the correlation between TRF users and the overall population size was not consistent over time; incidence rates after the label revision would be overestimated if TRF users increased more sharply than the overall population during 2013–2015. Fourth, data were available from only 10 states and might not be representative of the entire United States. Finally, data were available for only 3 years after the new label requirements took effect, and data were missing from four of the 10 states in 2014 or 2015. However, results and conclusions were essentially unchanged when sensitivity analyses were performed that used data from different groups of states (e.g., excluding states with missing data from analysis) and used different post-label periods (e.g., 2014 only and 2015 only). Nonetheless, the evaluation of the early impact from the Environmental Protection Agency’s intervention to reduce TRF-related illnesses should be considered preliminary and interpreted with caution.

Additional efforts are needed to prevent acute TRF-related illnesses, including promoting integrated pest management (*3*) to prevent and mitigate pest infestations and identifying more effective strategies to educate users about reading and following label instructions. Redesigning TRFs to prevent sudden, unexpected activation might also be useful.

**Summary**

**What is already known about this topic?**

Total release foggers (TRFs) pose a risk for acute illness. As a result, the Environmental Protection Agency required manufacturers to place improved labels on all TRFs manufactured after September 2012.

**What is added by this report?**

During 2007–2015, a total of 3,222 acute TRF-related cases were identified from 10 states participating in the Sentinel Event Notification System for Occupational Risk (SENSOR)–Pesticides program, the California Department of Pesticide Regulation program, and poison control centers in Florida, Texas, and Washington. No statistically significant reduction in overall incidence of TRF-associated injuries and illnesses was observed in the first 3 years after the label revisions took effect. Failure to vacate treated premises during application and early reentry of treated premises were the two most commonly reported causes of TRF-related illness. Failure to vacate the premises and excessive fogger use were associated with moderate or high severity illness.

**What are the implications for public health practice?**

More comprehensive strategies are needed to reduce acute TRF-related illnesses, including promoting integrated pest management and identifying better approaches for motivating users to read and follow label instructions. Redesigning TRFs to prevent sudden, unexpected activation might also be useful.

## Footnotes

* Under the SENSOR-Pesticides program, CDC provides cooperative agreement funding and technical support to state health departments to conduct surveillance for acute, occupational, pesticide-related illness and injury. Funding support is also provided by the Environmental Protection Agency. In 2017, a total of 13 states participated in this program. Data were available for this study for the period 2007–2013 in Oregon; 2007–2014 in California, Nebraska, and New York; and 2007–2015 in Florida, Louisiana, Michigan, North Carolina, Texas, and Washington. https://www.cdc.gov/niosh/topics/pesticides/overview.html.

† In California, two programs identify cases of acute pesticide-related illness and injury; one is located at the California Department for Public Health (CDPH) and participates in the SENSOR program; the other is the Pesticide Illness Surveillance Program (PISP), administered by CDPR. PISP operates similarly to the SENSOR program, but the case definition and the variables used to characterize cases differ between the two programs (http://www.cdpr.ca.gov/docs/whs/pisp.htm). Although PISP does not formally participate in the SENSOR program, both programs collaborate on joint activities. CDPH collects only work-related cases of acute pesticide-related illness and injury; PISP collects data for work-related and nonwork-related acute pesticide-related illness and injury. To ensure California cases were counted only once, CDPH cross-referenced its cases with those from PISP using name, date of illness and injury and, if available, Social Security number and date of birth.

§ 7,441 persons with TRF exposure were identified, and 3,222 unique cases remained after exclusions. The following persons were excluded: fewer than two signs or symptoms reported (3,638), suicide or intentional harm to others (24), and exposed to TRF products with unknown active ingredients (193). Also, 13 cases were reported by both the California SENSOR program and CDPR, and 351 by both SENSOR programs and PCCs in Florida, Texas, or Washington.

¶ In the SENSOR program, cases are defined as definite (objective evidence is available to confirm exposure and health effects), probable (a combination of objective and self-reported data), and possible (self-reported exposure and health effects data) (https://www.cdc.gov/niosh/topics/pesticides/pdfs/casedef.pdf). Cases from CDPR are categorized as definite (both physical and medical evidence documenting exposure and consequent health effects), probable (limited and circumstantial evidence supporting a relationship to pesticide exposure) and possible (health effects correspond generally to the reported exposure, but evidence is not available to support a relationship). http://apps.cdpr.ca.gov/calpiq/. Case categorization was not available for cases obtained only from PCCs in Florida, Texas, and Washington.

** Illness and injury severity for SENSOR and CDPR cases was categorized into four groups using the following standardized criteria: low severity (the condition usually resolves without treatment and <3 days are lost from work); moderate severity (the condition is not life-threatening but requires medical treatment; no residual impairment is expected, and time lost from work is ≤5 days); high severity (the condition is life threatening, requires hospitalization, often has >5 days lost from work, and might result in permanent impairment); and fatal (https://www.cdc.gov/niosh/topics/pesticides/statebase.html). For cases from PCCs in Florida, Texas, and Washington, case severity was based on the medical outcomes reported. Those cases reported with “death,” “major effect,” “moderate effect” and “minor effect” were classified as death, high severity, moderate severity, and low severity, respectively. PCC cases reported with “not followed, minimal clinical effects possible (no more than minor effect possible)” or “unable to follow, judged as a potentially toxic exposure” were also classified as low severity, unless, for those with “unable to follow, judged as a potentially toxic exposure,” if the call to the PCC arose from a health care facility and the case had at least two moderate or high severity signs or symptoms (https://www.cdc.gov/niosh/topics/pesticides/pdfs/pest-sitablev6.pdf), then the case was classified as moderate severity.
